# HoloPatch: improving intracardiac patch fit through holographically modelled templates

**DOI:** 10.1093/ehjimp/qyae103

**Published:** 2024-10-10

**Authors:** Matthias Lippert, Gabriella d’ Albenzio, Kathrine Rydén Suther, Karl-Andreas Dumont, Rafael Palomar, Hans Henrik Odland, Ole Jakob Elle, Bjørn Bendz, Henrik Brun

**Affiliations:** Division for Technology and Innovation, The Intervention Centre, Oslo University Hospital, PO Box 4950 Nydalen, 0424 Oslo, Norway; Institute of Clinical Medicine, University of Oslo, Kirkeveien 166, 0450 Oslo, Norway; Division for Technology and Innovation, The Intervention Centre, Oslo University Hospital, PO Box 4950 Nydalen, 0424 Oslo, Norway; Department of Informatics, University of Oslo, Oslo, Norway; Division of Radiology and Nuclear Medicine, Department of Radiology, Oslo University Hospital, Oslo, Norway; Department of Cardiothoracic Surgery, Oslo University Hospital, Oslo, Norway; Division for Technology and Innovation, The Intervention Centre, Oslo University Hospital, PO Box 4950 Nydalen, 0424 Oslo, Norway; Department of Computer Science, Norwegian University of Science and Technology, Gjøvik, Norway; Department of Paediatric Cardiology, Oslo University Hospital, Oslo, Norway; Department of Cardiology, Oslo University Hospital, Oslo, Norway; Division for Technology and Innovation, The Intervention Centre, Oslo University Hospital, PO Box 4950 Nydalen, 0424 Oslo, Norway; Department of Informatics, University of Oslo, Oslo, Norway; Institute of Clinical Medicine, University of Oslo, Kirkeveien 166, 0450 Oslo, Norway; Department of Cardiology, Oslo University Hospital, Oslo, Norway; Division for Technology and Innovation, The Intervention Centre, Oslo University Hospital, PO Box 4950 Nydalen, 0424 Oslo, Norway; Department of Paediatric Cardiology, Oslo University Hospital, Oslo, Norway

**Keywords:** mixed-reality, HoloLens, 3D print, congenital heart disease, cardiothoracic surgery

## Abstract

**Aims:**

Structural heart defects, including congenital ventricular septal defect closure or intracardiac rerouting, frequently require surgical reconstruction using hand-cut patch materials. Digitally modelled patch templates may improve patch fit and reduce outflow tract obstruction, residual defect risk, and conduction system damage. In this study, we benchmarked mixed-reality and a desktop application against a digitalized model of a real implanted patch.

**Methods and results:**

Ten patients scheduled for the repair of various defects consented to prospective inclusion in the study. After surgery, a digital model of the implanted patch was created from the residual material. Five clinical experts created 10 digital patches, 1 per patient, both in mixed-reality and desktop application, for comparison with the reference measurements, including the digitalized model of the real patch used during the surgery. Subjective residual shunt risk prediction was performed using both modalities. Digital patches created in mixed-reality closely matched the surgical material, whereas those created using desktop applications were significantly smaller. Different evaluators showed varying preferences for the application of the residual shunt risk and area.

**Conclusion:**

Digitally created patches can assist surgeons in preoperatively sizing of patch implants, potentially reducing post-operative complications.

## Graphical Abstract

**Graphical Abstract:**
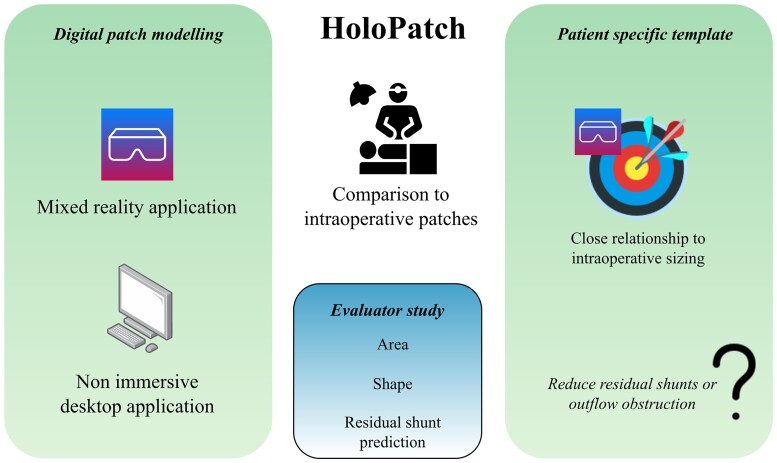

## Introduction

Reoperation due to stenosis or material failure has been observed in many patients with congenital heart disease years after surgery.^1^ Cardiac patch materials are most commonly used for ventricular septal defect (VSD) closure in congenital heart disease. Techniques for VSD closure vary depending on the defect type and location. The most common approach is open-heart surgery using autologous pericardium or Gore-Tex membrane, and sometimes direct suturing for smaller defects. Transcatheter device closure is less commonly employed. In more complex cardiac defects, a hole in the intraventricular septum can be used to redirect blood flow to a major vessel. Gore-Tex patches are typically used in intraventricular rerouting procedures, such as variants of the Rastelli procedure, for patients with transposition of the great arteries and/or double-outlet right ventricle, utilizing patches to redirect blood from one chamber through a VSD orifice into the correct artery.^2^ The incidence of residual shunts depends on how the shunt is defined and the detection methods used, such as intraoperative transoesophageal echocardiography or post-operative transthoracic echocardiography. The residual shunt rate after VSD closure ranges from 30 to 57%.^3^ Minor defects < 1 mm close spontaneously in 80% of cases, while defects > 2 mm show spontaneous closure in only one-third of cases.^4^ Residual shunts can result from small patches or due to anatomical factors. During the surgical placement of patches, it is crucial to avoid important structures such as the electrical conduction system and the aortic valve. However, the presence of tricuspid valve tissue, muscle bundles, or chordae attachments can complicate precise patch adaptation by interfering with the suture line.^5^ Conversely, oversized patches can cause outflow obstruction in 3–15% of patients with a double-outlet ventricle.^6,7^ Therefore, patch size is crucial. Sizing is based on intraoperative assessment, the surgeon’s experience, and imaging. For simple VSDs, preoperative planning typically involves 2D echocardiography. While 3D echocardiography enhances visualization by providing an *en face* view of the VSD shape, it is less commonly available. Olivieri *et al.*^8^ converted 3D raw echocardiographic data from nine VSD patients into 3D prints and found close similarities with 2D echocardiographic measurements. Perimembranous and muscular VSD morphologies vary, ranging from simple circular and oval shapes to tear drop-like defects.^9^ Complex VSDs are defined by several structures forming a virtual ring rather than a simple cut-out hole.^10^ Computed tomography (CT) and magnetic resonance imaging (MRI) are frequently used in cases of complex VSDs due to their superior spatial resolution. Although specialized software applications like 3D Slicer with the SlicerHeart extension are available for digital patch creation, their use in complex VSD closure remains limited.^11–13^ A patient-specific 3D-printed patch model could potentially improve the patch-to-patient fit during surgery. While mixed-reality and anatomic digital twin applications enhance depth perception, their accuracy has not been thoroughly studied. This study aimed to compare the accuracy of two methods for determining patch size and shape by analysing digital models from 3D Slicer desktop planning and real-time mixed-reality planning against a digitalized model of an actual surgical patch.

## Methods

### Patients

Ten patients planned for intracardiac patch procedures were selected based on the availability of preoperative CT imaging (*Table 1*). Pre-existing CT images were processed to create anatomical cardiac digital twins, with informed consent from the patient’s caregivers or parents. Patient-specific 3D models were created using a deep learning algorithm for cardiac CT image segmentation, as previously described.^14^ Following the artificial intelligence–derived segmentation output, the masks were manually edited using threshold-based painting and erasing to create a reference manual segmentation of the blood pool using 3D Slicer. The median segmentation time was 44 min [interquartile range (IQR) 28–55] per anatomic digital twin. The median time from CT imaging to surgery was 213 days (IQR 86–362), with an increase in body weight of 1.6 kg (IQR 1–2.6) and height of 4.5 cm (IQR 0–8).

**Table 1:** Overview of the patients

ID	Diagnose	Baffle task	Weight (kg)	Height (cm)
1	DORV TOF, doubly committed VSD	VSD rerouting	6.5	67
2	DORV TOF	VSD rerouting	11	78
3	DORV TOF	VSD rerouting	8.2	77
4	DORV TOF, absent pulmonary valve	VSD rerouting	75	172
5	PAPVD, double caval veins	Atrial baffle	3	52
6	DORV TOF, outlet extension	VSD rerouting	6	64
7	perimembranous VSD	VSD patch	6	61
8	muscular VSD	VSD patch	6	61
9	ccTGA, pulmonary atresia, Senning	Rastelli patch	10.3	77
10	TAC	VSD patch	2.4	46

ccTGA, congenitally corrected transposition of the great arteries; DORV, double-outlet right ventricle; PAPVD, partial anomalous pulmonary vein drainage; TAC, truncus arteriosus communis, TOF, tetralogy of Fallot; VSD, ventricular septal defect

### Gold standard digital reference patch creation

**Figure 1:**
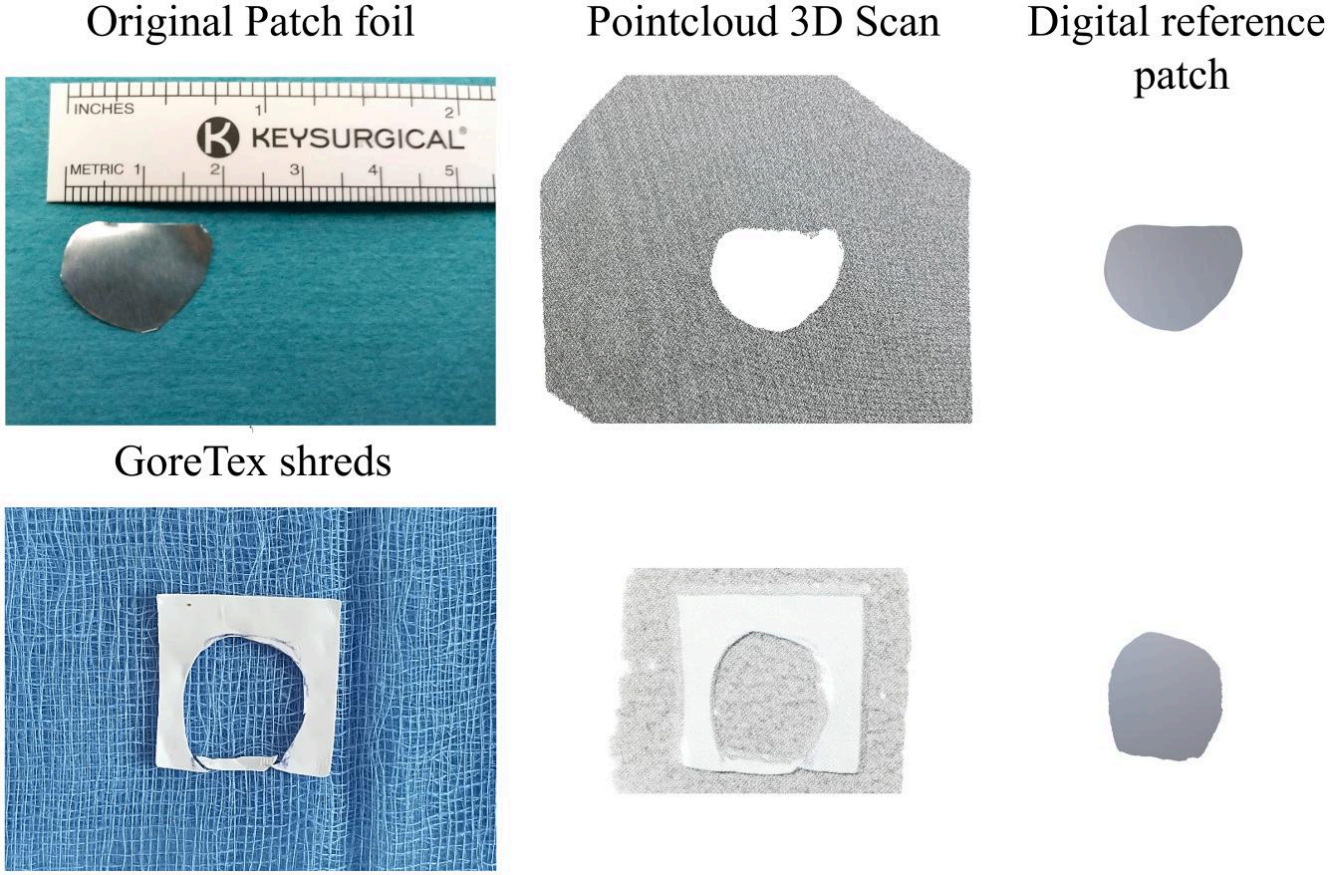
Creation of the DRP from physical object to digital object.

We created 10 digital patches based on intraoperative materials to serve as a standard for comparison. In six patients, we collected and reconstructed shreds of Gore-Tex material removed during surgery to assemble a negative patch. For the remaining four patients with autologous pericardial patches, we gathered the metal foils to which the pericardium was stapled during trimming to their shape and size (*Figure 1*). Subsequently, the materials were scanned using a 3D scanner (Revopoint Pop 2, Revopoint 3D Technologies Inc., China) with a 0.1 mm accuracy level to generate a point cloud. Data were processed using the open-source software CloudCompare (v2.12.4, www.cloudcompare.org) and Blender (v3.2.2, www.blender.org) to create a digital reference patch (DRP) (*Figure 1*).

### Radiologist measurements on CT data

**Figure 2:**
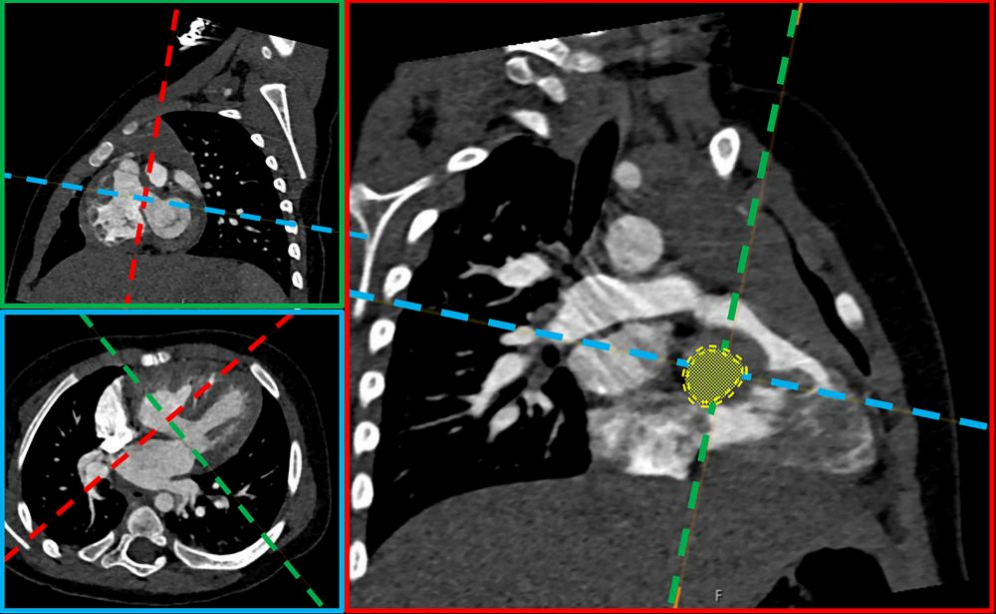
Illustration of VSD area measurement (yellow) calculation by radiologist.

Measurements were conducted by a senior radiologist with expertise in congenital heart disease CT examinations. Multiplanar reconstructions were performed to measure the area and diameter of the defect on a plane aligned with the ventricular septum and on two planes perpendicular to the VSD (*Figure 2*). Scan protocols were customized based on age, weight, and CT indications (*Table 2*). Imaging was performed on a third-generation dual-source CT system (Siemens SOMATOM Force, Erlangen, Germany) using high pitch mode, detector collimation of 2 × 192 × 0.6 mm, gantry rotation time of 250 ms, and automatic dose modulation. Contrast media Omnipaque 350 mg I/mL (GE Healthcare, USA) mixed with saline was administered and adjusted according to weight and indications. All patients, except one, received contrast medium in the right arm. A bi-phasic injection protocol was used in all paediatric patients, while a three-phase injection protocol was used for the adult patient. Bolus tracking in the ascending aorta was used to start the examination manually in paediatric patients and automatically with an ROI in the descending aorta for the adult. All examinations, except one, were performed without electrocardiographic gating.

**Table 2:** Overview of the imaging details of each patient

ID	Cardiac phase^a^	Sedation	ECG gating	Injection arm site	contrast amount (mL)	Flow (mL/s)	Injection protocol^b,c^	DLP (mGycm)	CTDIvol (mGy)^c^
1	Systole	+	−	Right	5	0.8	10 mL 50%, 10 mL NaCl	12	0.73
2	Systole	+	+	Right	8.5	1.1	15 mL 50%, 10 mL NaCl	36	1.89
3	Systole	+	−	Right	6.5	1	13 mL 50%, 9 mL NaCl	17	0.85
4	Systole	−	−	Right	92.5	5.6	75 mL 100%, 35 mL 50%, 30 NaCl	92	2.4
5	Diastole	−	−	Right	4.5	0.7	9 mL 50%, 9 mL NaCl	9	0.56
6	Systole	+	−	Right	4.5	0.8	9 mL 50%, 8 mL NaCl	15	0.87
7	Systole	+	−	Left	8	1	12 mL 70%, 9 mL NaCl	16	0.74
8	Systole	+	−	Left	8	1	12 mL 70%, 9 mL NaCl	16	0.74
9	Systole	+	−	Right	10	0.8	12 mL 80%, 14 mL NaCl	27	1.1
10	Diastole/systole	−	−	Right	4	0.6	7 mL 50%, 7 mL NaCl	7	0.45

^**a**^Cardiac phase: in the non–ECG-gated exams, the position of the valves and the distension of the cardiac chambers were evaluated to estimate in which part of the cardiac cycle the exam was performed

^b^Injection protocol: in all, but one patient, a bi-phasic injection protocol was used. First a mixture of contrast media and saline was given (the percentage annotates the amount of contrast media) and this was followed by a saline chase. In the grown up individual, the first bolus was pure contrast media, followed by a contrast media/saline mix, and finally a saline chase

^c^Phantom size used for CTDIvol was 32 cm

### Mixed-reality application

Participants used the *Draw Patch tool* from the TruHeart App H.S.1.0.0 (Holocare, Oslo, Norway) on a Microsoft HoloLens 2 (Microsoft, Redmond, WA, USA) for mixed-reality modelling (see Supplementary data online, *Video Appendix*). The digital patch creation process involves marking the circumference of the desired patch on a segmented heart model using a sphere on the fingertips of the user’s avatar hand. The system collects collision points where the sphere overlaps with the heart model. Once marked, the user activates the patch creation feature. The algorithm interpolates these points to form a ring conforming to the geometry of the heart. The midpoint of this ring was calculated, allowing the user to adjust the curvature of the patch on the plane created using a midpoint marker. This adjustment updates the Bézier curve interpolation and finalizes the design. After solidification, the template can be exported as an *STL file* for 3D printing. A k-d tree algorithm enhances the interface responsiveness by locating arbitrary points in the 3D model.^15^

### Desktop application

An alternative set of patches was created using the SlicerHeart^12^ extension in 3D Slicer.^11^ For each patient, a scene was prepared with the heart walls opened to provide a clear view of the anatomy, avoiding the extended learning period required to master 3D view manipulation in 3D Slicer. The user examines the defects from various perspectives. On average, each expert evaluator placed a minimum of 10 initial points, referred to as anchor points, to outline the boundary curve of the patch. These anchor points were adjustable on the mesh surface after initial placement to ensure perfect adherence to the 3D heart model. After the final adjustments were made, the scene was saved for subsequent analysis.

### Expert evaluation study

Five expert evaluators (two congenital heart surgeons and three senior cardiologists with extensive experience in intraoperative echocardiographic guidance) created digital patches using the HoloLens 2 or desktop application. Clinician tasks ranged from a priori categorized from standard VSD closure (Patients 7 and 8) to moderately difficult tasks such as double-outlet right ventricle patch rerouting (Patients 1–4, 6, and 10) and demanding tasks with atrial baffle and Rastelli patches (Patients 5 and 9). The selection was based on the intention to test the feasibility of the applications in different use-case scenarios. The task order was randomized for each session and user. HoloLens sessions were completed first because of limited access to software licences. Between the evaluation by HoloLens and desktop application, there was a median washout period of 47 *±* 15 days. Both methods were performed in the same room under identical ambient lighting conditions. Users were given 10 min to familiarize themselves with the applications before starting. For each patient, evaluators quantified the risk of the residual shunt on a scale of 0–5 and indicated potential regions of shunt related to the finished patch. A change of > 90° was considered a relevant change in the residual shunt position.

### Statistical analysis

Digital patches generated using both methods were analysed for area differences and assessments of Gaussian curvatures using the Visualisation Toolkit (VTK) and its standard filters to calculate the areas and curvatures of patches. The results were validated using various methods. The distribution of continuous variables was graphically assessed. Following a normal distribution, Student’s *t*-test was applied. The absolute area difference between the HoloLens or desktop application–based patches and DRP was calculated for all patients and evaluators. The mean absolute difference between all evaluators and patients was used for the Student’s *t*-test. All data are expressed as medians and IQR unless otherwise specified. Standard deviations of the patch areas and diameters were averaged from the values of the five evaluators for each method and patient. All statistical analyses and graphics were performed using STATA (version 18.0; StataCorp LLC, TX, USA).

## Results

### Area measurements

All area calculations are summarized in *Table 3*. We compared the mean absolute difference between all patches in the comparison of HoloLens vs. DRP, with a 95% confidence interval of (*−*141, 72) mm^2^, and between the desktop application and DRP, with a 95% confidence interval of (−241, −75) mm^2^. Given these confidence intervals, we observed a significant difference in the patches created in the desktop application to the DRP rather than in the complementary absolute difference between HoloLens and DRP. The following factors describe the relationship between the area measurements obtained using different methods.


$$\frac{HoloLens}{DRP}=0.84(\mathrm{IQR}0.6-1.6)$$

$$\frac{Desktop\;application}{DRP}=0.58(\mathrm{IQR}0.4-1.2)$$

$$\frac{HoloLens}{Desktop\;application}=1.42(\mathrm{IQR}1.25-1.54)$$

**Table 3:** Comparison of area (mm^2^ between DRP gold standard, CT, and the median area of digitally created patches by each method

ID	DRP	CT	Desktop application	HoloLens	Residual shunt
1	255	86	170 ± 17	249 ± 46	0
2	196	113	300 ± 22	383 ± 64	1
3	300	133	238 ± 25	324 ± 60	1
4	725	887	1017 ± 107	1544 ± 206	0
5^a^	934		423 ± 44	562 ± 107	0
6	74	74	81 ± 7	105 ± 43	0
7	187	32	30 ± 5	44 ± 16	1
8	53	65	44 ± 16	147 ± 35	0
9	1181	193	468 ± 40	650 ± 86	0
10	221	93	107 ± 9	154 ± 12	0

^**a**^Direct pre-operative measurement of atrial baffle not possible in CT

**Figure 3:**
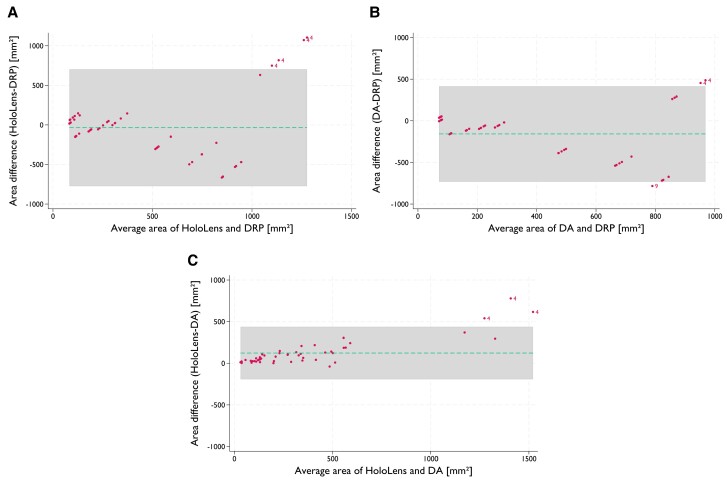
Bland–Altman plots for the area (mm^2^) for *A*) HoloLens vs. digital reference patch (DRP), *B*) Desktop application (DA) vs. DRP, and *C*) HoloLens vs. desktop application. Note: Points outside the limits are labelled by patients’ numbers.

All participants created larger digital patches in HoloLens than in the desktop application, with slight variations between the evaluators [P3 1.15 × (IQR 1–1.39), P4 1.76 × (IQR 1.5–1.9)]. The different methods are compared in *Figure 3*. The results of the Bland–Altman plots revealed a mean area difference (*DRP −HoloLens*) = *−*35 mm^2^, (*DRP − Desktop app*) = *−*158 mm^2^, and (*HoloLens − Desktop app*) = 124 mm^2^.

### Diameter measurements

To determine the diameters of the patches, we applied principal component analysis (PCA) to reduce the data set to its two principal components.^16^ We then estimated the diameters along these axes, and the results are presented in *Table 4*. For comparison with standard clinical evaluations, *Table 4* also includes pre-operative echocardiographic and CT-derived measurements, along with intraoperative descriptions.

**Table 4:** Comparison of diameters (mm) between CT, considered the gold standard, and PCA-derived diameters from DRP and digitally created patches by each method

ID	CT	Pre-operative echocardiography description	DRP	Desktop application	HoloLens	Intraoperative description
1	15 × 7	9 × 8	27 × 12	18 *±* 1 × 12 *±* 1	18 *±* 1 × 16 *±* 1	
2	12 × 8	10 × 10	19 × 14	20 *±* 1 × 18 *±* 1	23 *±* 1 × 21 *±* 1	30 × 25
3	18 × 9	14	21 × 19	21 *±* 1 × 15 *±* 1	23 *±* 1 × 17 *±* 1	20 × 15
4	30 × 24	25	33 × 29	39 *±* 2 × 33 *±* 1	44 *±* 3 × 38 *±* 1	40 × 30
5^a^	23	—	37 × 32	25 × 17 *±* 1	28 *±* 1 × 18 *±* 2	25 diameter
6	9 × 6	5	11 × 9	11 *±* 1 × 9	13 *±* 2 × 11 *±* 1	8
7	5 × 3	5 × 4	10 × 8	7 × 6 *±* 1	8 *±* 1 × 7 *±* 1	15 × 10
8	8 × 6	9 × 6	17 × 15	16 *±* 1 × 8	17 *±* 2 × 10 *±* 2	5
9	16 × 12	15 × 12	47 × 32	28 *±* 1 × 21 *±* 1	28 *±* 1 × 23 *±* 1	
10	12 × 10	14 × 9	20 × 16	14 × 9 *±* 1	15 × 12 *±* 1	30 × 25

^a^Direct pre-operative measurement of atrial baffle not possible in CT or echocardiography

### Curvature analysis

**Figure 4:**
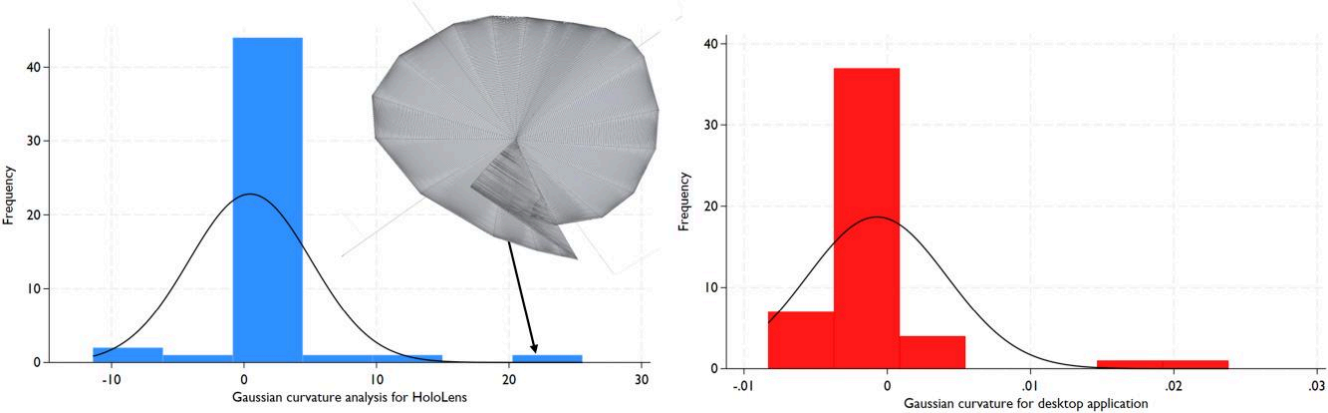
Histograms for Gaussian curvature by method (HoloLens, blue; desktop application, red). Note the five patches with values amplified by factor 10^3^.

**Figure 5:**
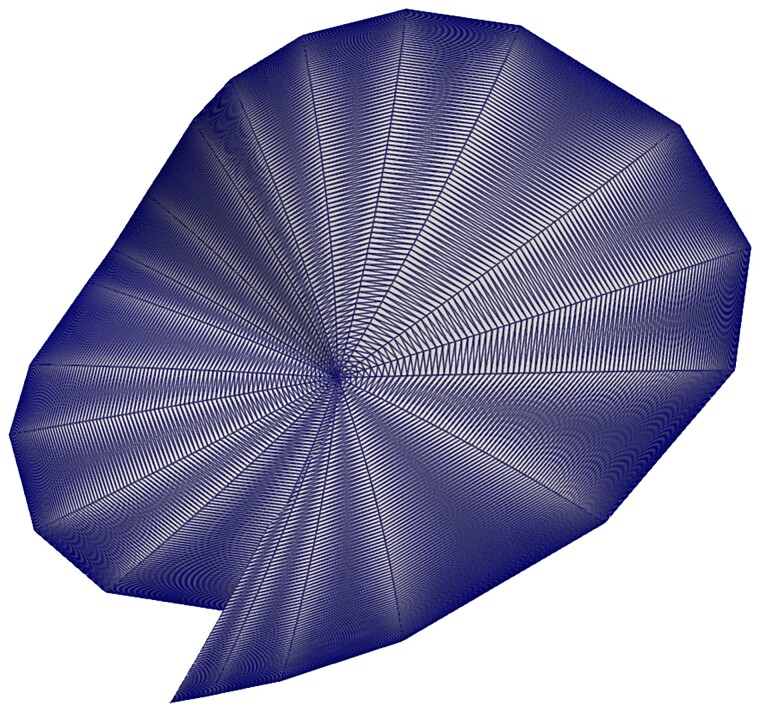
Self-intersection of mesh produced in HoloLens at 7 o’clock.

The mean Gaussian curvature describes the shape of the patch, where positive values indicate a dome-like shape and negative values describe a saddle-like shape. The median value for HoloPatch was −0.0045, with an IQR of −0.0134 to 0.004. For desktop applications, the median was −0.0015, with an IQR ranging from −0.003 to 0 (*Figure 4*). However, we encountered five patches where malformations were registered in the HoloLens method, which appeared owing to the inaccuracy of the circumferential ring generation, causing a self-intersection or ‘double fold’ in the geometry (*Figures 4* and *5*).

### Risk assessment

**Figure 6:**
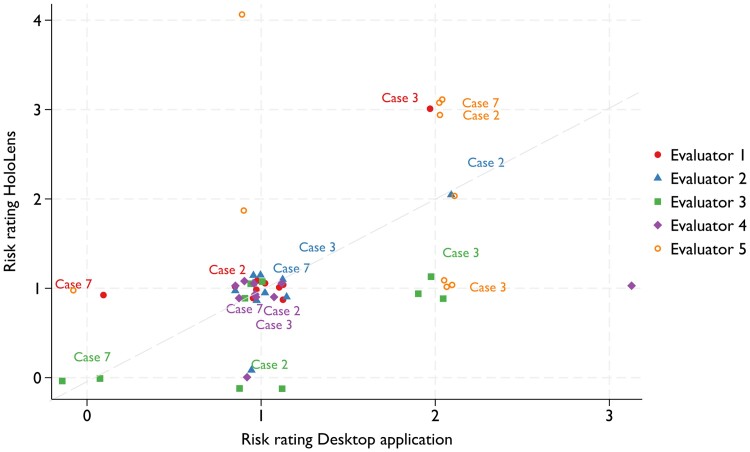
Jitter scatterplot showing risk assessment for residual shunt for HoloLens (*y*-axis) and desktop applications (*x*-axis). Note: Patients 2, 3, and 7 with residual shunts.

The risk assessment results for residual shunts using HoloLens and desktop applications are shown in *Figure 6*. The ratings were visualized using random jitter to improve the visualization of overlapping data points. The predicted position of the residual shunt changed in five patients for three evaluators, in four patients for one evaluator, and in one patient for one evaluator when assessed with different modalities. Post-operative echocardiograms were used to locate the position of the residual shunt, which has been registered in 3 out of 10 patients in our collective and used to compare the position indicated by the expert evaluators on the 3D model. Notably, in the three patients with residual shunts, two out of five evaluators correctly identified the position of the residual shunt when evaluating the patients’ anatomy with HoloLens. One evaluator better identified the position of the residual shunt with the desktop application; for the remaining evaluators, the correct position was not predicted.

## Discussion

This study found that mixed-reality–based VSD patch models closely resembled implanted patches, whereas desktop application–based models had smaller areas. HoloLens patches were slightly more curved, primarily due to application-related artefacts. The evaluators had varied subjective assessments of possible residual shunts and their positions, with no clear advantage for either method compared to the surgical results.

First, the real-time designed HoloLens patch models were closer in size to the intraoperative findings than those obtained using the desktop application. The feasibility of extended reality or 3D print applications for pre-operative simulation has been demonstrated for the closure of various VSD types.^17–19^ Contrary to these studies, the present study is the first to report a real-time interactive patch design performed entirely in mixed-reality with a direct comparison to intraoperative findings, as represented by DRP. Mixed-reality technology enables the evaluation of anatomy with a different depth perception compared to other modalities, such as desktop application volume rendering or traditional tomographic slices. HoloLens-designed patches had a small *−*35 mm^2^ (*Figure 3A*) difference from the DRP, while desktop application patches showed a mean −158 mm^2^ (*Figure 3B*) area difference. It is challenging to determine whether this difference is primarily due to immersive user experience or other factors. Additional mechanisms could include less precise placement of the planned suture line using a fixed-size fingertip sphere in the HoloLens application compared to adjustable anchor points in the desktop application (see Supplementary data online, *Video Appendix*); nevertheless, this leads to a result that remains closer to the real patch used and a preference for this application. Residual shunt rates range from 30 to 57%, comparable with our study, with 3 of 10 patients having post-operative residual shunts confirmed using post-operative echocardiography. Notably, in Patients 2 and 3, HoloLens-derived values indicated under-sizing of the intraoperatively used VSD patch, potentially contributing to residual shunts and highlighting the potential benefits of this mixed-reality application.

Secondly, we observed differences in the shapes of the digital patches between the HoloLens and desktop application methods. The HoloLens application has limitations in accurately marking the patch circumference on the heart model. This was owing to the use of a sphere at the user’s fingertip to define the patch edges, with the system collecting the collision points. However, the 3D model’s nature and hand positioning may have led to inaccuracies in point detection, causing steps due to marking in a more proximal adjacent plane than the intended plane in the model and the need for repetition of the process. Additionally, collision points are non-adjustable compared with the anchor points of desktop application. These inaccuracies contribute to the formation of self-intersections in mixed-reality (*Figures 4* and *5*). Although we were aware of this phenomenon before the study, we could not prevent it from occurring in five patches. *Post hoc* analysis demonstrated that this phenomenon only affected the curvature analysis but did not significantly impact the area results. Four patches resulted in a single self-intersection with <than 10% change in area, whereas in only one patch, there was a 30% area change due to a double self-intersection. Another technical aspect is the difference in computational restrictions compared to HoloLens 2 with a Snapdragon 850 Mobile CPU and a desktop 16 GB GPU utilized in this study. Cubic Bézier surfaces have been employed in collaborative virtual reality environments for surgical planning because of their ability to facilitate rapid manipulation of surfaces.^20^ Mixed-reality applications are often rendered using a HoloLens CPU. Intricately detailed heart models comprising numerous vertices combined with manipulation of Bézier surfaces might hinder user experience. However, these technologies can enhance the clinical value of repeatedly simulating complex suture lines prior to surgery.

Thirdly, risk evaluation of the residual shunt results compared to actual post-operative residual shunts demonstrated a range of outcomes. *Figure 6* suggests that different evaluators may benefit from different modalities. Evaluators 1 and 4 correctly identified a higher risk of residual shunts for Patients 3 and 7 and 2 and 7, respectively. Conversely, using the desktop application, Evaluators 3 and 4 correctly identified a higher risk in Patients 2 and 3 and 3, respectively. It was difficult to draw a causal interpretation from these results; however, we suggest that different applications may be more suitable for different users depending on their preferences, as both methods were new to the evaluators in this study.

This study had some limitations. The small sample size limits conclusions regarding causality or statistically significant results. Moreover, real-time patch modelling is complex and depends on several variables to obtain accurate results. As shown in *Table 2*, most scans were performed during the systolic phase based on the valve positions in the CT scan. ECG-gated CT imaging involves higher radiation exposure; therefore, it is avoided, unless necessary, for diagnostic purposes in children. *Table 3* shows the differences between the DRP and HoloLens patches. The differences are possibly because of the non-physiological relaxation and dilatation of the structures in cardioplegia during heart surgery. Depending on the position of the VSD, this might equally affect the patch size for both methods. This affects defects in the muscular septum more than those in the fibrotic perimembranous septum with extension, of which the latter was more common in our study (*n* = 7). Several studies have shown accurate defect measurements with both non–ECG-gated and prospectively gated CT acquisition.^21–24^ Engagingly, Oliveri *et al.* achieved a correlation of 0.988 between 3D printing and standard 2D echocardiographic measurements using transthoracic 3D echocardiography data, warranting further use of this radiation-free imaging modality.^8^ However, owing to the heart rate, the 3D models were created based on four or five frames of data, which may have reduced their accuracy. State-of-the-art mini 3D transoesophageal probes may optimize the frame rate and improve image quality; however, they are mainly used intraoperatively and not for pre-operative planning. A workflow combining CT at the diagnostic stage with precise sizing updates from 3D echocardiography directly before surgery could counteract possible changes in defect size during the delay to surgery, which is a clear limitation of our study. The heart models in this study lacked myocardium, which could affect patch sizing. Finally, DRP attempts to replicate the intraoperative patch as closely as possible. Human pericardium processed with Carpentier’s or glutaraldehyde solution exhibits unique anisotropic properties, making it unlike stretchable based on longitudinal or transverse insertion.^25^ Moreover, smaller intraoperative shreds were not accounted for after the initial adjustment of the foil, which was used for comparison. These limitations did not affect the Gore-Tex–derived DRP; however, reassembling multiple shreds in these patches might have affected their diameters, as shown in *Table 4*. The combination of imaging acquisition considerations, timing from imaging to surgery, and evaluation of surgically trimmed patches are limitations of our results and should be studied further to advance the *in vivo* application of the workflow.

## Conclusion

Real-time interactive patches created in mixed-reality showed a higher correlation with intraoperatively used materials than those created using a non-immersive application. Creating templates for intraoperative use requires further standardization and evaluation of image acquisition, 3D modelling, and methodological computational aspects.

## Supplementary data

Supplementary data are available at *European Heart Journal - Imaging Methods and Practice* online.

## Supplementary Material

qyae103_Supplementary_Data

## Data Availability

The data sets generated and/or analysed during the current study are not publicly available due to institutional privacy policies but are available from the corresponding author on reasonable request with relevant research and established data-sharing agreements.

## Lead author biography

Matthias Lippert, MD, graduated from Goethe University Frankfurt, Germany. He is currently a research fellow at the Division for Technology and Innovation at Oslo University Hospital and working at Akershus University Hospital as a cardiology resident. His main areas of interest are 3D imaging and advanced visualization methods by utilizing artificial intelligence.

## References

[1] Peivandi AD, Martens S, Asfour B, Martens S. Grafts and patches: optimized but not optimal materials for congenital heart surgery. Pediatr Cardiol 2023;44:996–1002. 37038028 10.1007/s00246-023-03153-6 PMC10224861

[2] Lecompte Y, Vouhé P. Réparation à l'Etage ventriculaire (REV procedure): not a Rastelli procedure without conduit. Oper Tech Thorac Cardiovasc Surg 2003;8:150–59.

[3] Bibevski S, Ruzmetov M, Mendoza L, Decker J, Vandale B, Jayakumar KA et al The destiny of postoperative residual ventricular septal defects after surgical repair in infants and children. World J for Pediatr Congenit Heart Surg 2020;11:438–43. 10.1177/2150135120918537 32645789

[4] Dodge-Khatami A, Knirsch W, Tomaske M, Prêtre R, Bettex D, Rousson V et al Spontaneous closure of small residual ventricular septal defects after surgical repair. Ann Thorac Surg 2007;83:902–05. 17307430 10.1016/j.athoracsur.2006.09.086

[5] Andersen HØ, de Leval MR, Tsang VT, Elliott MJ, Anderson RH, Cook AC. Is complete heart block after surgical closure of ventricular septum defects still an issue? Ann Thorac Surg 2006;82:948–56. 16928514 10.1016/j.athoracsur.2006.04.030

[6] Kim CY, Kim WH, Kwak JG, Jang WS, Lee CH, Kim DJ et al Surgical management of left ventricular outflow tract obstruction after biventricular repair of double outlet right ventricle. J Korean Med Sci 2010;25:374–79. 20191035 10.3346/jkms.2010.25.3.374 PMC2826730

[7] Oladunjoye O, Piekarski B, Baird C, Banka P, Marx G, Nido D et al Repair of double outlet right ventricle: midterm outcomes. J Thorac Cardiovasc Surg 2020;159:254–64. 31597616 10.1016/j.jtcvs.2019.06.120

[8] Olivieri LJ, Krieger A, Loke YH, Nath DS, Kim PCW, Sable CA. Three-dimensional printing of intracardiac defects from three-dimensional echocardiographic images: feasibility and relative accuracy. J Am Soc Echocardiogr 2015;28:392–97. 25660668 10.1016/j.echo.2014.12.016

[9] McCarthy KP, Leung C, Ho PK, Y S. Perimembranous and muscular ventricular septal defects —morphology revisited in the era of device closure. J Interv Cardiol 2005;18:507–13. 16336433 10.1111/j.1540-8183.2005.00093.x

[10] Yim D, Dragulescu A, Ide H, Seed M, Grosse-Wortmann L, van Arsdell G et al Essential modifiers of double outlet right ventricle: revisit with endocardial surface images and 3-dimensional print models. Circ Cardiovasc Imaging 2018;11:e006891. 29855425 10.1161/CIRCIMAGING.117.006891

[11] Fedorov A, Beichel R, Kalpathy-Cramer J, Finet J, Fillion-Robin JC, Pujol S et al 3D slicer as an image computing platform for the quantitative imaging network. Magn Reson Imaging 2012;30:1323–41. 22770690 10.1016/j.mri.2012.05.001 PMC3466397

[12] Lasso A, Herz C, Nam H, Cianciulli A, Pieper S, Drouin S et al SlicerHeart: an open-source computing platform for cardiac image analysis and modeling. Front Cardiovasc Med 2022;9:886549. 36148054 10.3389/fcvm.2022.886549 PMC9485637

[13] Vigil C, Lasso A, Ghosh RM, Pinter C, Cianciulli A, Nam HH et al Modeling tool for rapid virtual planning of the intracardiac baffle in double-outlet right ventricle. Ann Thorac Surg 2021;111:2078–83. 33689734 10.1016/j.athoracsur.2021.02.058 PMC8154721

[14] Nainamalai V, Lippert M, Brun H, Elle OJ, Kumar RP. Local integration of deep learning for advanced visualization in congenital heart disease surgical planning. Intell Based Med 2022;6:100055.

[15] Bentley JL . Multidimensional binary search trees used for associative searching. Commun ACM 1975;18:509–17.

[16] Pearson K . LIII. On lines and planes of closest fit to systems of points in space. London Edinburgh Dublin Philos Mag J Sci 2010;2:559–72.

[17] Aljassam Y, Caputo M, Biglino G. Surgical patching in congenital heart disease: the role of imaging and modelling. Life (Basel) 2023;13:2295. 38137896 10.3390/life13122295 PMC10745019

[18] Liang J, Lu B, Zhao X, Wang J, Zhao D, Zhang G et al Feasibility analyses of virtual models and 3D printing for surgical simulation of the double-outlet right ventricle. Med Biol Eng Comput 2022;60:3029–40. 36053430 10.1007/s11517-022-02660-7

[19] Mendez A, Hussain T, Hosseinpour AR, Valverde I. Virtual reality for preoperative planning in large ventricular septal defects. Eur Heart J 2019;40:1092. 30329041 10.1093/eurheartj/ehy685

[20] Chheang V, Bruggernann R, Preim B, Hansen C, eds. Virtual resection planning using Bezier surface interactions in collaborative VR environments. *2023 IEEE Conference on Virtual Reality and 3D user interfaces abstracts and workshops (VRW);* Shanghai, China, 2023-03.

[21] Isobe S, Katayama Y, Ozawa T, Fujii T. Intracardiac three-dimensional image as surgical decision-making tool of congenital heart disease. Pediatr Cardiol 2024;45:351–60. 38017199 10.1007/s00246-023-03349-w

[22] Ma XJ, Tao L, Chen X, Li W, Peng ZY, Chen Y et al Clinical application of three-dimensional reconstruction and rapid prototyping technology of multislice spiral computed tomography angiography for the repair of ventricular septal defect of tetralogy of Fallot. Genet Mol Res 2015;14:1301–09. 25730069 10.4238/2015.February.13.9

[23] Nau D, Wuest W, Rompel O, Hammon M, Gloeckler M, Toka O et al Evaluation of ventricular septal defects using high pitch computed tomography angiography of the chest in children with complex congenital heart defects below one year of age. J Cardiovasc Comput Tomogr 2019;13:226–33. 30737152 10.1016/j.jcct.2019.01.023

[24] Olejník P, Nosal M, Havran T, Furdova A, Cizmar M, Slabej M et al Utilisation of three-dimensional printed heart models for operative planning of complex congenital heart defects. Kardiol Pol 2017;75:495–501. 28281732 10.5603/KP.a2017.0033

[25] Tremblay D, Zigras T, Cartier R, Leduc L, Butany J, Mongrain R et al A comparison of mechanical properties of materials used in aortic arch reconstruction. Ann Thorac Surg 2009;88:1484–91. 19853098 10.1016/j.athoracsur.2009.07.023

